# Errata

**Published:** 2011-12

In the Abstract of their article “Estimating Water Supply Arsenic Levels in the New England Bladder Cancer Study” [Environ Health Perspect 119:1279–1285 (2011)], Nuckols et al. reported the following results:

Three methods accounted for 93% of the residential estimates of arsenic concentration: direct measurement of water samples (27%; median, 0.3 μg/L; range, 0.1–11.5), statistical models of water utility measurement data (49%; median, 0.4 μg/L; range, 0.3–3.3), and statistical models of arsenic concentrations in wells using aquifers in New England (17%; median, 1.6 μg/L; range, 0.6–22.4). The authors have revised the measurements using a more accurate method for calculating the median (weighted by person-years) and for reporting the range (25th–95th percentile) based on the values reported in Table 1 of the article, which are correct. The revised measurements are as follows:

Three methods accounted for 93% of the residential estimates of arsenic concentration: direct measurement of water samples (27% EY; median weighted by person-years = 0.3 µ µg/L; 25–95th percentile range: 0.1–20.7 µg/L), statistical models of water utility measurement data (49% EY; weighted median 0.4 µg/L; range, 0.2–3.8 µ µg/L), and statistical models of arsenic concentrations in wells using aquifers in New England (17% EY; weighted median: 1.7 µ µg/L; range, 0.5–30.5 µg/L).

The revisions do not change the study’s primary results, discussion, or conclusions. Nowhere else in the article is the range in concentration by water supply source summarized by broad source categories.

In the article by Balazs et al. [ Environ Health Perspect 119:1272–1278 (2011)], Equation 1 was incorrect. The corrected equation appears below.


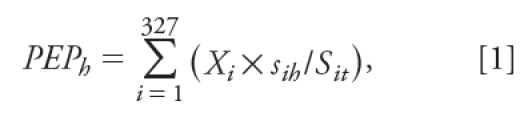
[1]

*EHP* apologizes for the error.

